# Skin Microstructure is a Key Contributor to Its Friction Behaviour

**DOI:** 10.1007/s11249-016-0794-4

**Published:** 2016-11-30

**Authors:** Maria F. Leyva-Mendivil, Jakub Lengiewicz, Anton Page, Neil W. Bressloff, Georges Limbert

**Affiliations:** 1 National Centre for Advanced Tribology at Southampton (nCATS), Faculty of Engineering and the Environment, University of Southampton, Southampton, SO17 1BJ UK; 2 Bioengineering Science Group, Faculty of Engineering and the Environment, University of Southampton, Southampton, SO17 1BJ UK; 3 Institute of Fundamental Technological Research, Polish Academy of Sciences (IPPT PAN), ul. Pawinskiego 5B, 02-106 Warsaw, Poland; 4 Biomedical Imaging Unit, Faculty of Medicine, University of Southampton, Southampton General Hospital, Southampton, SO16 6YDJ UK; 5 Computational Engineering and Design Group, Faculty of Engineering and the Environment, University of Southampton, Southampton, SO17 1BJ UK; 6 Laboratory of Biomechanics and Mechanobiology, Division of Biomedical Engineering, Department of Human Biology, Faculty of Health Sciences, University of Cape Town, Observatory, Cape Town, 7935 South Africa

**Keywords:** Skin, Friction mechanisms, Contact mechanics, Microstructure, Finite element, Image-based modelling, Material properties

## Abstract

Due to its multifactorial nature, skin friction remains a multiphysics and multiscale phenomenon poorly understood despite its relevance for many biomedical and engineering applications (from superficial pressure ulcers, through shaving and cosmetics, to automotive safety and sports equipment). For example, it is unclear whether, and in which measure, the skin microscopic surface topography, internal microstructure and associated nonlinear mechanics can condition and modulate skin friction. This study addressed this question through the development of a parametric finite element contact homogenisation procedure which was used to study and quantify the effect of the skin microstructure on the *macroscopic* skin frictional response. An anatomically realistic two-dimensional image-based multilayer finite element model of human skin was used to simulate the sliding of rigid indenters of various sizes over the skin surface. A corresponding structurally idealised multilayer skin model was also built for comparison purposes. Microscopic friction specified at skin asperity or microrelief level was an input to the finite element computations. From the contact reaction force measured at the sliding indenter, a homogenised (or apparent) macroscopic friction was calculated. Results demonstrated that the naturally complex geometry of the skin microstructure and surface topography alone can play as significant role in modulating the deformation component of macroscopic friction and can significantly increase it. This effect is further amplified as the ground-state Young’s modulus of the *stratum corneum* is increased (for example, as a result of a dryer environment). In these conditions, the skin microstructure is a dominant factor in the deformation component of macroscopic friction, regardless of indenter size or specified local friction properties. When the skin is assumed to be an assembly of nominally flat layers, the resulting global coefficient of friction is reduced with respect to the local one. This seemingly counter-intuitive effect had already been demonstrated in a recent computational study found in the literature. Results also suggest that care should be taken when assigning a coefficient of friction in computer simulations, as it might not reflect the conditions of microscopic and macroscopic friction one intends to represent. The modelling methodology and simulation tools developed in this study go beyond what current analytical models of skin friction can offer: the ability to accommodate arbitrary kinematics (i.e. finite deformations), nonlinear constitutive properties and the complex geometry of the skin microstructural constituents. It was demonstrated how this approach offered a new level of mechanistic insight into plausible friction mechanisms associated with purely structural effects operating at the microscopic scale; the methodology should be viewed as complementary to physical experimental protocols characterising skin friction as it may facilitate the interpretation of observations and measurements and/or could also assist in the design of new experimental quantitative assays.

**Electronic supplementary material:**

The online version of this article (doi:10.1007/s11249-016-0794-4) contains supplementary material, which is available to authorized users.

## Introduction

Besides its multiple physiological functions as the largest organ of the human body [1], the skin is essentially a complex mechanical interface separating and protecting the internal body structures from the external environment. As humans go through their life, their skin is constantly subjected to mechanical contact interactions with a wide range of objects and devices which include clothing fabrics, footwear, seating and bedding surfaces, sports equipment, personal care products (e.g. razor, skin care lotion) or medical devices, not to mention intra- and interindividual skin-to-skin interactions [2–4]. These tribological interactions are an essential part of how humans perceive their environment whether it is for cognitive awareness, social interaction or self-preservation. This is achieved through the ability of the skin to act as a multiphysics sensory interface which converts physical stimuli (e.g. deformation, temperature, presence of noxious chemical substances) into a neural response relayed to the brain. These physical stimulations are sensed by an elaborate network of sensory receptors embedded within the skin [5, 6]. When the skin mechanically interacts with an external surface through contact, its surface and underlying microstructure can undergo temporary or permanent deformations sufficient to activate sensory receptors. These, in turn, trigger action potentials by converting mechanical energy into electrochemical energy. Ionic currents are then generated and propagated through nerve fibres to ultimately reach the brain cortex. Therefore, the load transmission process from an external surface to the skin external surface and deeper internal microstructure is critical in how mechanically induced discomfort and pain are engendered [7].

Skin friction, which is manifested as forces resisting the motion of skin relative to other surfaces, is a complex phenomenon which conditions and, at the same time, is part of this load transmission process. Understanding the physical mechanisms that give rise to skin friction is therefore essential in furthering our knowledge of it and in developing novel solutions and improved products that are optimally designed to interact with the skin. The corollary aspect of discomfort and pain which are evolutionary survival mechanisms is that excessive mechanical loading can lead to damage, and, eventually, to loss of structural integrity of the skin (e.g. skin tears, friction blisters, pressure ulcers). Evidence suggests that friction mechanisms are the key in these damage processes [8–11].

Although in the last decade skin friction has attracted a significant interest and a large body of work [2, 4, 7, 12–36], to date, a unifying theory that encompasses the interaction of skin with counter surfaces or even defines the dominant contributing parameters is still not available. The main factor limiting the development of predictive models is that skin–object interaction is a highly nonlinear and multifactorial system [31, 33]. The parameters that affect the interaction behaviour of skin encompass the geometrical, mechanical and biophysical domains and, next to application-related interaction parameters such as contact pressures and sliding velocities, include the local microclimate (temperature and humidity) as well as individual’s characteristics (e.g. age, ethnicity and sex).

Of particular relevance to skin tribology in general, and skin friction in particular, is the intra-individual natural variability of the mechanical properties of the *stratum corneum*—the outermost layer of the skin consisting of a 15–20-cell-thick self-renewable layer of keratinised epithelial cells [37]. Modifications of external environmental conditions such as humidity level can significantly alter the stiffness of the *stratum corneum* [22, 38, 39]: Wu et al. [39] reported a Young’s modulus of 0.6 and 370 MPa, for 100 and 30% relative humidity (RH). Such variations in mechanical properties have significant effects on the distribution of strains in the subjacent layers, as demonstrated in a recent anatomically based computational study by Leyva-Mendivil et al. [40]. Changes in the *stratum corneum* stiffness also influence the direct macroscopic structural response of the skin to various types of loading conditions. Moreover, the plasticising effect of high humidity on the *stratum corneum* leads to its softening which is accompanied by an increase in real area of contact and therefore adhesion, resulting in an increase in the skin frictional response [20, 36, 39, 41]. This effect can lead to a greater likelihood of mechanical damage to the skin in the form of superficial pressure ulcers and friction blisters [2, 11, 42–44] or skin tears [9].

The friction responses of soft materials involve the contribution of both an adhesion and a deformation component [45]. The adhesion component is directly linked to the notion of real area of contact (sum of microasperity contact areas), while the deformation component is associated with the geometry and deformations of asperities that resist the relative motion of the contacting surfaces. In the literature, authors rather talk about an adhesion and a *hysteresis* component of friction [46, 47]. This seems to imply that time-dependent and/or inelastic effects arising through viscous dissipation are necessary to provide a contribution to friction. This is not the case as the presence of asperities and their associated *elastic* deformations are sufficient to induce mechanical resistance (i.e. forces) against a slider. By consequence, we think it is more appropriate to refer to a *deformation* component of friction be it elastic or inelastic.

In solid mechanics, it is often assumed that surface roughness (*i.e.* geometric characteristics of surface topography at a small scale) of materials is a main contributor to friction [48]. It was shown by Stupkiewicz et al. [49] that the geometrical effects alone can have a significant impact on the macroscopic frictional response of elastic contacts. Despite this, only a few studies have investigated the contribution of the skin micromesoscopic topography to its global friction response [2]. These experimental studies showed contradicting or inconclusive results: Egawa et al. [50] indicated that the skin surface roughness, even though not correlated with skin friction, improved the predictability of the coefficient of friction when analysed along skin moisture in multiregression analyses; Nakajima and Narasaka [24] showed that the density of the skin primary furrows is correlated with skin friction, but also found correlation between furrow density and elasticity; however, it is unclear which of these factors dominates the skin friction response [2]. A detailed overview of our current understanding of skin friction can be found in recent seminal papers [2, 7, 12, 23, 27, 33]. In most of these studies, the topographic features of the skin are assumed to provide negligible or no contribution to the skin global friction response, because of the high compliance of the skin compared to that of the indenter. However, on the one hand, it is reasonable to assume that the existence and distortion of the skin topographic features during sliding contact could significantly contribute to the skin global friction response [51]. On the other hand, because skin is often subjected to wetting conditions, the frictional effects due to elastohydrodynamic lubrication could play a significant role.

The topography of the skin is dependent on age and body location [2, 19, 34] and so are its mechanical and bio-physico-chemical properties. The unknown nonlinear interplay between these factors is what makes the study of skin friction so difficult. These aspects are implicitly captured—but not separated and quantified—in physical tribological experiments measuring skin friction. These measurements are often reported as macroscopic friction calculated from the reaction force obtained from the relative motion of a surface with respect to the skin [23]. Only few studies report the skin friction response measured at a microscopic scale: Pailler-Mattei et al. [26] measured the coefficient of friction of isolated *stratum corneum* with a 7.8-μm-radius spherical diamond indenter, and Tang and Bhushan [28] analysed the coefficient of friction for single-asperity contact provided by an etched Si probed of 10 nm radius on murine skin.

Macroscopic values of coefficient of friction between the skin and various materials are often those used as *input* in computational studies simulating skin friction [11, 52, 53]. If the dimensions of these models are consistent with macroscopic spatial scales, this modelling assumption is legitimate. However, if some parts of the models feature different spatial scales, this assumption might be questionable. This observation is also an opportunity to formulate and develop mechanistic hypotheses about the nature of the relationship between *microscopic friction* response at asperity level, hereafter referred as *local friction*, and *macroscopic friction* (hereafter referred as *global friction)*.

In the study of skin friction, a number of questions arise. Is skin microrelief a potentially significant contributor to macroscopic skin friction? Can variations in the mechanical properties of the *stratum corneum* affect the role of skin surface topography in modulating macroscopic friction? To date, and to the best of the authors’ knowledge, no study has addressed these questions using a physics-based finite element quantitative approach which is the main aim of the study reported in this paper. Here, we explored the role of the skin surface topography and internal microstructure on its global friction response. This was achieved by means of a two-dimensional anatomically based finite element model of human skin [40] interacting with rigid indenters of various sizes. A second idealised multilayer skin model, representing a nominally flat surface, was used for comparison purposes. The sliding of these indenters (that should be more precisely referred as *sliders*) over the skin surface was simulated. Local coefficients of friction between the skin and indenter were also varied. The (macroscopic) contact reaction forces experienced by these indenters during sliding were measured to determine an equivalent macroscopic coefficient of friction which was then compared to the applied local coefficient of friction. At this stage, and very importantly, it is worth pointing out that the rigid sliders considered in the computational analyses could be viewed as single asperities of a macroscopic flat rigid surface.

The paper is organised as follows. In Sect. 2, the general modelling methodology including the characteristics of the skin models and the design of computer experiments are described. This section also describes the post-processing procedure to calculate equivalent macroscopic friction coefficients. This approach can be viewed as a computational homogenisation technique. The results of the simulations are described in Sect. 3 and discussed in Sect. 4 while final concluding remarks are provided in Sect. 5.

## Modelling Methodology

In this study, finite element techniques were applied for the computational simulation of skin contact interactions with rigid bodies. This approach allowed quantifying of the contribution of the skin topography and microstructure deformations to the global friction response for various contact scenarios. A series of coefficients of friction at a local scale was used for the representation of different contacting materials and/or equivalent local contact interface properties. Variation of the *stratum corneum* stiffness was performed to simulate the hardening/softening effects of different humidity conditions. Furthermore, the effects of different asperity dimensions (represented by the indenter radius) were assessed in order to identify possible structural effects of contact interactions at microscopic and macroscopic scales. Here, and in the rest of this paper, with a slight abuse of language, the term *microscopic* refers to sub-millimetric dimensions.

### Contact Sampling and Averaging Procedure

A recent micromechanical computational study by Stupkiewicz et al. [49] quantified the role of asperity geometry on the observed macroscopic anisotropic friction of rough surfaces. Their approach consisted of generating random micro‐topographies of surfaces, applying periodic boundary conditions, assigning a local coefficient of friction, applying macroscopic loading conditions to induce a sliding motion and measuring the resultant global contact forces. In order to derive an equivalent (or macroscopic) coefficient of friction, spatial, time and ensemble averaging was applied; therefore, the method was extremely time consuming. In the present work, a computationally more efficient, albeit simplified, method for averaging the macroscopic frictional response was applied for the problem of a macroscopically flat skin sliding against a macroscopically flat rigid surface. Both of these macroscopically flat surfaces contain microasperities which contribute to the sliding resistance between the surfaces. The main simplifying assumption and hypothesis of this work is that the *microscopically rough* rigid surface was made of randomly positioned identical cylindrical indenters. The anatomical geometry of the skin model provided the microscopic asperities in the form of crests and furrows which are part of its topography. A single two-millimetre-long skin sample was used in this study, assuming that it was that of a representative geometry. The indenters (i.e. asperities of the rigid surface) were assumed to be located sufficiently far from each other so that their mutual interference to the local contact interactions at the skin surface was negligible. Based on the above assumptions, a representative microsample consisting of the skin sample in contact with a single indenter can be used to derive the global friction response of the macroscopically flat surfaces with the averaging procedure described below. (see Fig. 3). The indenter position was given with respect to the *undeformed* skin sample; however, the full sliding contact problem was analysed in the *deformed* configuration.

The macroscopic normal (vertical) and tangential (horizontal) components of the traction vector are $\bar{f}_{\text{N}} = \sum\nolimits_{i} {f_{\text{N}}^{i} }$ and $\bar{f}_{\text{T}} = \sum\nolimits_{i} {f_{\text{T}}^{i} }$, respectively, where $f_{\text{N}}^{i}$ and $f_{\text{T}}^{i}$ are total contact reaction forces at the *i*th asperity (indenter). If the number of asperities is large enough, these forces can be replaced by their respective integral representations, i.e.

1 $$\bar{f}_{N} \simeq \frac{\rho L}{{D_{x} }}\int_{x = 0}^{{D_{x} }} {f_{N} } (x)dx,\quad\, \bar{f}_{T} \simeq \frac{\rho L}{{D_{x} }}\int_{x = 0}^{{D_{x} }} {f_{T} } (x)dx$$

where *x* is the horizontal position of the indenter, *D*_*x*_ is the sliding distance over the nominal width of the skin microsample and *L* is the macroscopic length of the rough surface. The quantity *ρ* is the average number of indenters per unit length (indenters’ density).

Our assumptions enabled the use of a simplified procedure to calculate the macroscopic frictional response from the solution of a single microscopic contact problem. The cylindrical rigid indenter was pressed down and slid over the skin sample, as depicted in Fig. 3. The reaction forces experienced by the rigid slider were sampled at different vertical positions *x* ^*j*^ of the slider along the sliding path. Finally, by applying the trapezoidal integration rule, the macroscopic reaction forces could be recovered as:

2 $$\bar{f}_{N} \simeq \frac{\rho L}{{D_{x} }}\sum\limits_{j} {\frac{1}{2}} (x^{j} - x^{j - 1} )(f_{N} (x^{j} ) + f_{N} (x^{j - 1} )),$$

3 $$\bar{f}_{T} \simeq \frac{\rho L}{{D_{x} }}\sum\limits_{j} {\frac{1}{2}} (x^{j} - x^{j - 1} )(f_{T} (x^{j} ) + f_{T} (x^{j - 1} )),$$

and, after simplifications, the *macroscopic* or *global* coefficient of friction was obtained as:

4 $$\mu_{g} = \frac{{\bar{f}_{T} }}{{\bar{f}_{N} }} \simeq \frac{{\sum\nolimits_{j} {(x^{j} - x^{j - 1} )} (f_{T} (x^{j} ) + f_{T} (x^{j - 1} ))}}{{\sum\nolimits_{j} {(x^{j} - x^{j - 1} )} (f_{N} (x^{j} ) + f_{N} (x^{j - 1} ))}}.$$

### Multilayer Finite Element Models of the Skin

**Fig. 1:**
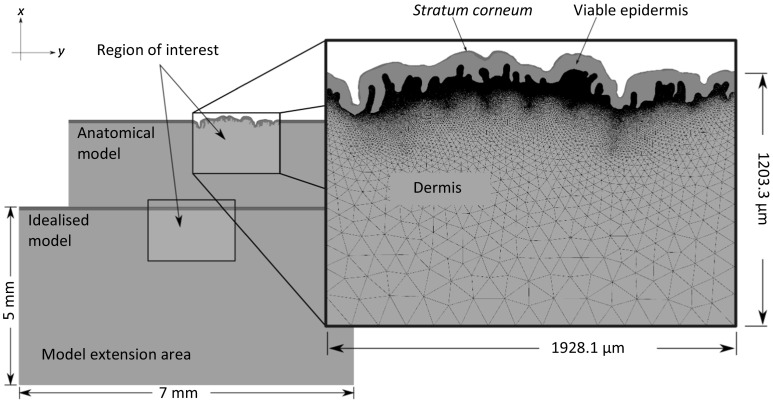
Skin models. The anatomical (*top*) and idealised (*bottom*) skin models were appropriately dimensioned to avoid boundary effects in the finite element analyses, according to the recommendations by Karduna et al. [54]. The detailed plane strain mesh of the anatomically based skin model is shown, indicating the dimensions of the region of interest. To enhance visibility, the edges of the finite elements making up the *stratum corneum* and viable epidermis are not shown

The skin was modelled in 2D using a plane strain assumption and the geometry of the *anatomical* model based on histological sections of a mid-back skin sample obtained from a healthy 30-year-old Caucasian female with no known medical conditions. The procedures for sample preparation, image acquisition, image segmentation and finite element meshing are provided in Leyva-Mendivil et al. [40]. The model considered the intricate geometry of the skin topography and that of the different layer interfaces, identifying the *stratum corneum*, viable epidermis and dermis. However, in the present study, the two internal skin layers were assumed to have the same mechanical properties and, therefore, could have been modelled as a single layer. The effect of distinct mechanical properties for the dermis and viable epidermis could be explored in future studies. The segment of skin previously considered in the anatomical skin model [40] was set as what we call the *region of interest* in the present study (see Fig. 1). The interactions on this section are representative of a single asperity (i.e. the rigid slider) of a macroscopically flat rigid surface. The dimensions of the skin model were expanded outside this area according to the recommendations of Karduna et al. [54] to avoid boundary effects in the contact simulations. In order to be able to isolate the effects of the skin microstructure (including external surface topography and interlayer topography) by way of comparison, a *geometrically idealised* skin model was built. This model took into account the mean thickness of the *stratum corneum* and viable epidermis from the anatomical model to provide an *idealised* representation of the skin, as a flat multilayered tissue (see Fig. 1). The finite element meshes of the idealised and anatomical models were generated within the finite element environment of Abaqus 6.14 (Simulia, Dassault Systèmes, Providence, RI, USA). The meshes were exported to the symbolic/numeric AceGen/AceFEM package [55] integrated within Mathematica (Wolfram Research, Inc., Champaign, IL, USA.) for the finite element simulation of the skin contact interactions. The characteristic element size in the idealised model varied from 2 μm at the *stratum corneum* to 150 μm at the base of the region of interest, resulting in 151,127 linear triangular elements. In order to capture the irregular geometry, further mesh refinement was required in the anatomical model where the minimum element size in the *stratum corneum* was 1.5 μm leading to a total of 336,224 elements for the whole skin model.

Following the approach taken in Leyva-Mendivil et al. [40], skin layers were modelled using a neo-Hookean hyperelastic strain energy potential:

5 $$\psi = c_{10} (\bar{I}_{1} - 3) + \frac{{\kappa_{0} }}{2}(J - 1)^{2}$$

defined with the first deviatoric invariant of the right Cauchy–Green deformation tensor ${\mathbf{C}}$, $\bar{I}_{1} = J^{{ - \frac{2}{3}}} ({\mathbf{C}}:{\mathbf{I}})$ where the volume ratio $J = \sqrt {\det ({\mathbf{C}})}$ provides a measure of material compressibility and ${\mathbf{I}}$ is the second-order identity tensor. The parameters *c*_10_ and *κ*_0_ correspond to half the shear modulus and bulk modulus of an isotropic linear elastic material, respectively. At small strains, neo-Hookean elasticity is equivalent to isotropic linear Hookean elasticity [56], so that *c*_10_ and *κ*_0_ can be expressed as functions of the Young’s modulus *E* and Poisson’s ratio *ν*:

6 $$c_{10} = \frac{E}{{4\left( {1 + \nu } \right)}}\;{\text{and}}\;\kappa_{0} = \frac{E}{{4\left( {1 - 2\,\nu } \right)}}$$

### General Contact Modelling Approach

For the experimental characterisation of skin friction, it is required to impose relative motion of the skin and contacting surface to generate a reaction or traction force. Most experiments use load cells oriented in the indenting and sliding direction to measure the normal and tangential components of this traction vector [23]. The ratio of these components determines the global coefficient of friction they report. In the literature, most skin friction studies consider relatively large surfaces (an indenter, a roller or a flat surface), reporting values of *macroscopic* friction. In contrast, only few studies report the skin friction response at a microscopic scale [26, 28]. In the present study, it is proposed to compare the microscopic (or local) friction response of skin to the macroscopic (or global) friction for the same contacting materials and environmental conditions. In the finite element analyses to be described below, local friction will be an *input* parameter while global friction will be an *output* response calculated from the traction vector by the post-processing of results generated from the contact simulations.

#### Contact Formulation

Contact of deformable bodies with rigid cylindrical indenter is a standard problem, even for the finite deformation regime which introduces additional geometrical non-linearities. In the present work, the contact interaction was defined by a local coefficient of friction *μ*_*l*_. The contact unilateral constraints were regularised with an augmented Lagrangian technique and implemented within AceGen/AceFEM system, applying the approach developed in Lengiewicz et al. [57]. The standard contact framework developed for the quasi-static regime was not sufficient to assure convergence of the microscopic skin contact problem. The difficulty was due to the complexity of the skin surface topography which induced highly nonlinear snap-through and snap-back phenomena. In order to overcome these convergence problems and to stabilise the solution, the standard Newmark integration scheme was applied [58]. This approach effectively boils down to adding dynamical terms absent from the quasi-static formulation to the elastic model of the skin. The Newmark scheme parameters and velocities were adjusted such that the influence of the applied stabilisation on the solution was negligible.

#### Mechanical Properties

The mechanical properties of the dermis and viable epidermis were assumed to be identical and constant for all the finite element simulations: *E*_*D*_ = *E*_*VE*_ = 0.6 MPa and *ν*_*D*_ = *ν*_*VE*_ = 0.3 [59–61].

#### Fixed Boundary Conditions

The 2D skin models were contained within a (*x*, *y*) plane where the *x*-axis is parallel to the mean contact surface and the *y*-axis is orthogonal to it. A rigid discoidal indenter of variable radius was modelled to simulate contact interactions with the skin. Prior to any finite element analysis, it was positioned on top of the centre of the region of interest, so that indentation was performed along the direction of the *y*-axis, and sliding along the direction of the *x*-axis (see Fig. 1). The base of each skin model (defined by *y* = 0) was rigidly fixed.

#### Indentation Displacement Conditions

**Fig. 2:**
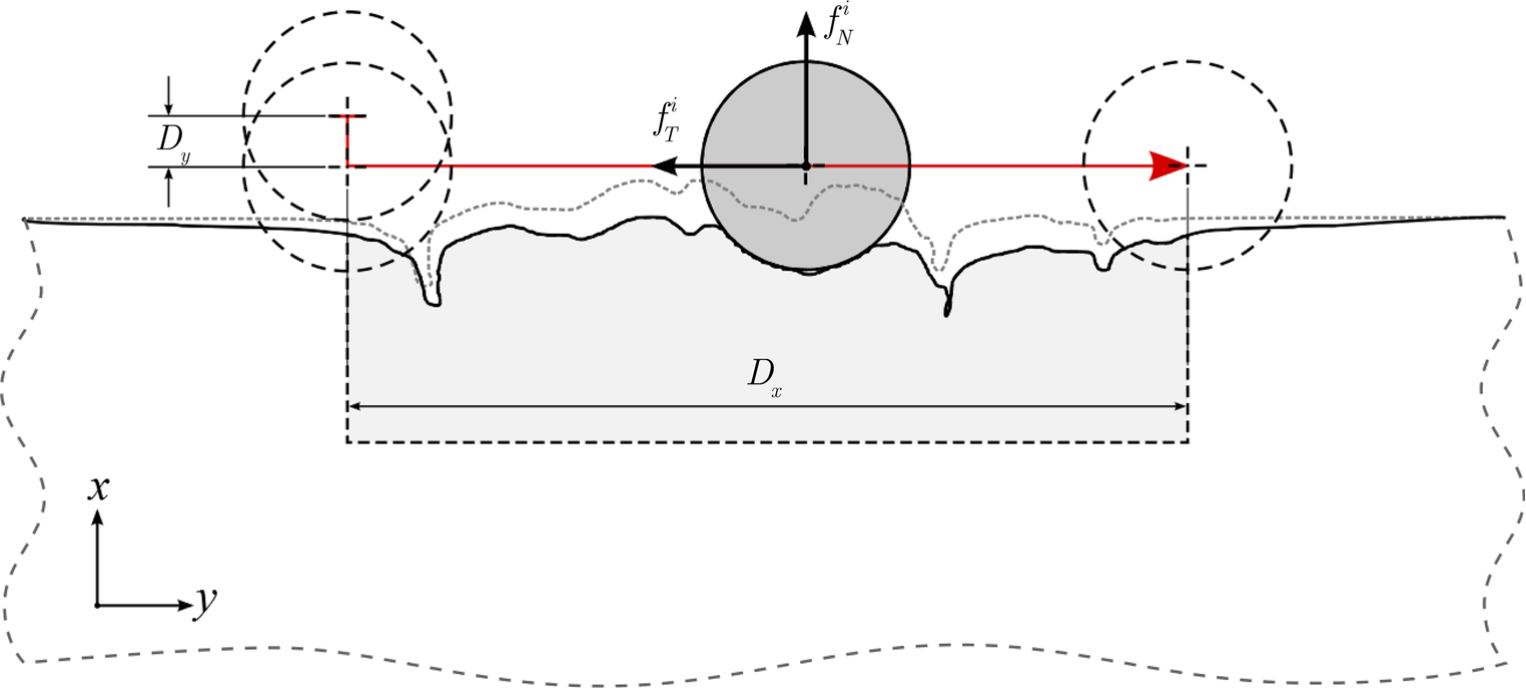
Illustration describing the simulation steps. *Step 1* Indentation of the skin surface is simulated with the application of a vertical displacement of magnitude *D*_*y*_ to the indenter. *Step 2* Sliding of the rigid indenter over the skin surface is simulated with the application of a horizontal displacement of magnitude *D*_*x*_ to the indenter, resulting in a global reaction force whose components *f*_*N*_^*i*^ and *f*_*T*_^*i*^ are used to calculate the global coefficient of friction. The *grey dashed line* indicates the undeformed geometry (*i.e*. initial conditions) while the *solid outlines* represent the current deformed geometry (*i.e.* an intermediate step of the simulation). The *red arrow* indicates the full trajectory that the indenter follows (Color figure online)

**Fig. 3:**
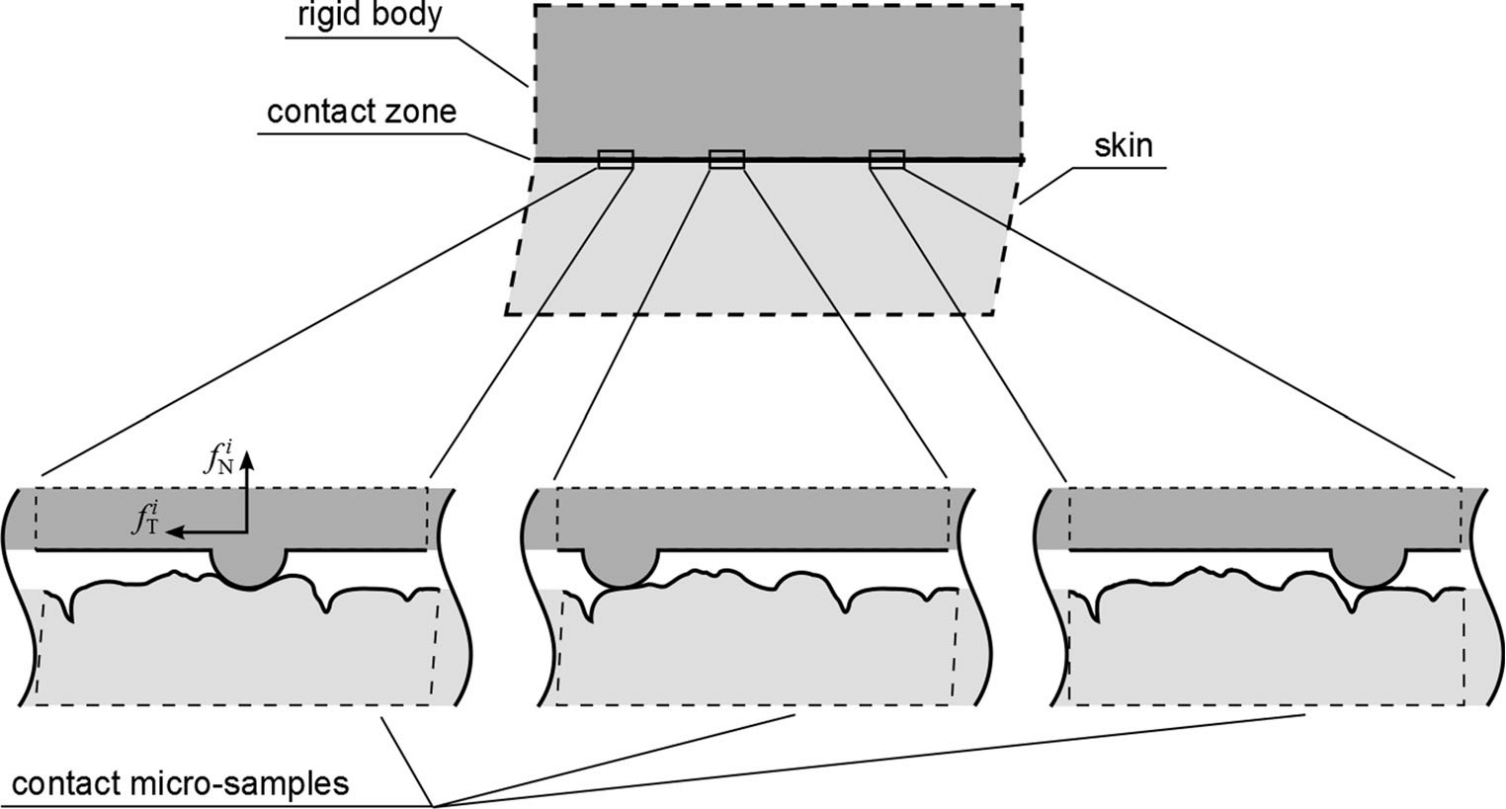
Conceptual illustration of frictional contact of an idealised rigid rough surface with the skin. *Zoomed-in views* (*bottom*): each asperity of the rigid surface can be idealised as a discoidal rigid indenter

The indentation step was defined by imposing a *D*_*y*_ displacement to the indenter along the *y*-axis direction. In order to avoid boundary effects, the indenter displacement was set to *D*_*y*_ = *R*_1_/2 for microscale contact [54] (see Fig. 2). In the anatomical model, the displacement was set with respect to its nominal height, so that its deformation was equivalent to that of the idealised skin model (Fig. 3).

#### Combined Indentation and Sliding Displacement Conditions

The analysis was conducted in two steps: first, a pure vertical indentation was applied, followed by a horizontal displacement of the indenter while maintaining the initial vertical displacement. To enforce stability of contact analyses, low intensity viscous forces (with negligible effects on the solution) were added to the contact formulation. For this reason, once the maximum indentation displacement *D*_*y*_ was reached, a stabilisation period was allowed prior to the beginning of the sliding step (second step). The sliding motion was set towards the right vertical edge of the model (see Figs. 1, 2).

### Analysis Variants

In order to represent various contact interaction scales, three indenter dimensions were considered, setting the radius of the indenter *R*_1_ to 0.1, 0.25 and 0.5 mm. Additionally, with a view to investigate the softening effects of relative humidity on the *stratum corneum* in relation to macroscopic friction, two sets of mechanical properties were considered for the *stratum corneum*, each corresponding to a distinct relative humidity level: (*E*_*SC*_ = 0.6 MPa, *ν*_*SC*_ = 0.3) and (*E*_*SC*_ = 370 MPa, *ν*_*SC*_ = 0.3), respectively, at 100 and 30% relative humidity. These values of Young’s modulus were measured by Wu et al. [39] while the choice of the Poisson’s ratio value was based on previous studies [40, 62]. Four values of local coefficient of friction, *μ*_*l*_, were considered: 0.0 (*i.e.* frictionless contact), 0.1, 0.2 and 0.3. For each combination of skin model type (idealised or anatomical), analysis type (indentation or indentation combined to sliding motion), indenter radius, Young’s modulus of the *stratum corneum* and local coefficient of friction a unique finite element analysis was conducted resulting in a total of 48 analyses. All the values of varying model parameters considered in this study are listed in Table 1.

**Table 1:** Values of material, geometrical and system properties considered in the design of computer experiment applied to the study of contact interaction for the idealised and anatomical models of skin and indenter

Parameter	Symbol	Values
Young’s modulus of *stratum corneum*	*E*_*SC*_	0.6, 370 MPa
Indenter radius	*R*_1_	0.1, 0.25, 0.5 mm
Local coefficient of friction	*μ*_*l*_	0, 0.1, 0.2, 0.3

The verification of the computational *idealised* skin models was performed by comparing the finite element results to relevant corresponding analytical models described in Online Resource provided with this manuscript.

## Results

The simulation featuring the following combination of parameters [*E*_*SC*_ = 0.6 MPa, *R*_1_ = 0.50 mm, *μ*_*l*_ = 0.3] could not converge before completion of the whole sliding distance. In order to estimate the global coefficient of friction that could not be calculated from the finite element results, a quadratic regression of the form *μ*_*g*_(*μ*_*l*_) = *a* * *μ*_*l*_^2^ + *μ*_*l*_ was established from the results of fully converged simulations featuring the same combination of *E*_*SC*_ and *R*_1_. A regression equation exhibiting a coefficient of determination *R* ^2^ = 0.9946 was obtained for *a* = −0.02926. A summary of the sliding distances and global friction results is provided in Table 2.

**Table 2:** Global coefficients of friction as a function of the Young’s modulus of the *stratum corneum*, indenter size an local coefficients of friction for both idealised and anatomical models

Analysis			Idealised		Anatomical	
*E*_*SC *_[MPa]	*R*_1_ [mm]	*μ*_*l*_	Sliding distance [mm]	*μ*_*g*_	Sliding distance [mm]	*μ*_*g*_
0.6	0.1	0	1.937	0.000	2.010	0.007
0.6	0.25	0	1.929	0.000	2.010	0.003
0.6	0.5	0	1.959	0.000	2.009	0.001
0.6	0.1	0.1	1.983	0.076	2.013	0.130
0.6	0.25	0.1	2.003	0.088	2.010	0.109
0.6	0.5	0.1	2.021	0.091	2.010	0.100
0.6	0.1	0.2	1.999	0.157	2.010	0.259
0.6	0.25	0.2	2.010	0.176	2.010	0.216
0.6	0.5	0.2	1.995	0.181	2.008	0.199
0.6	0.1	0.3	1.988	0.237	2.002	0.395
0.6	0.25	0.3	2.010	0.265	2.010	0.325
0.6	0.5	0.3	2.022	0.269	1.121	0.297^a^
370	0.1	0	1.993	0.000	2.015	0.034
370	0.25	0	1.966	0.000	2.005	0.002
370	0.5	0	1.973	0.000	2.010	0.001
370	0.1	0.1	1.997	0.069	2.019	0.148
370	0.25	0.1	2.001	0.088	2.007	0.111
370	0.5	0.1	1.996	0.096	2.005	0.101
370	0.1	0.2	1.170	0.157	2.004	0.291
370	0.25	0.2	1.996	0.181	2.009	0.225
370	0.5	0.2	2.002	0.194	2.010	0.206
370	0.1	0.3	0.882	0.258	2.009	0.388
370	0.25	0.3	1.001	0.278	2.011	0.343
370	0.5	0.3	1.980	0.291	2.005	0.310

^a^Value estimated with quadratic regression of *μ*_*g*_(*μ*_*l*_) for *R*_1_ = 0.5 mm and *E*_*SC*_ = 0.6 MPa

**Fig. 4:**
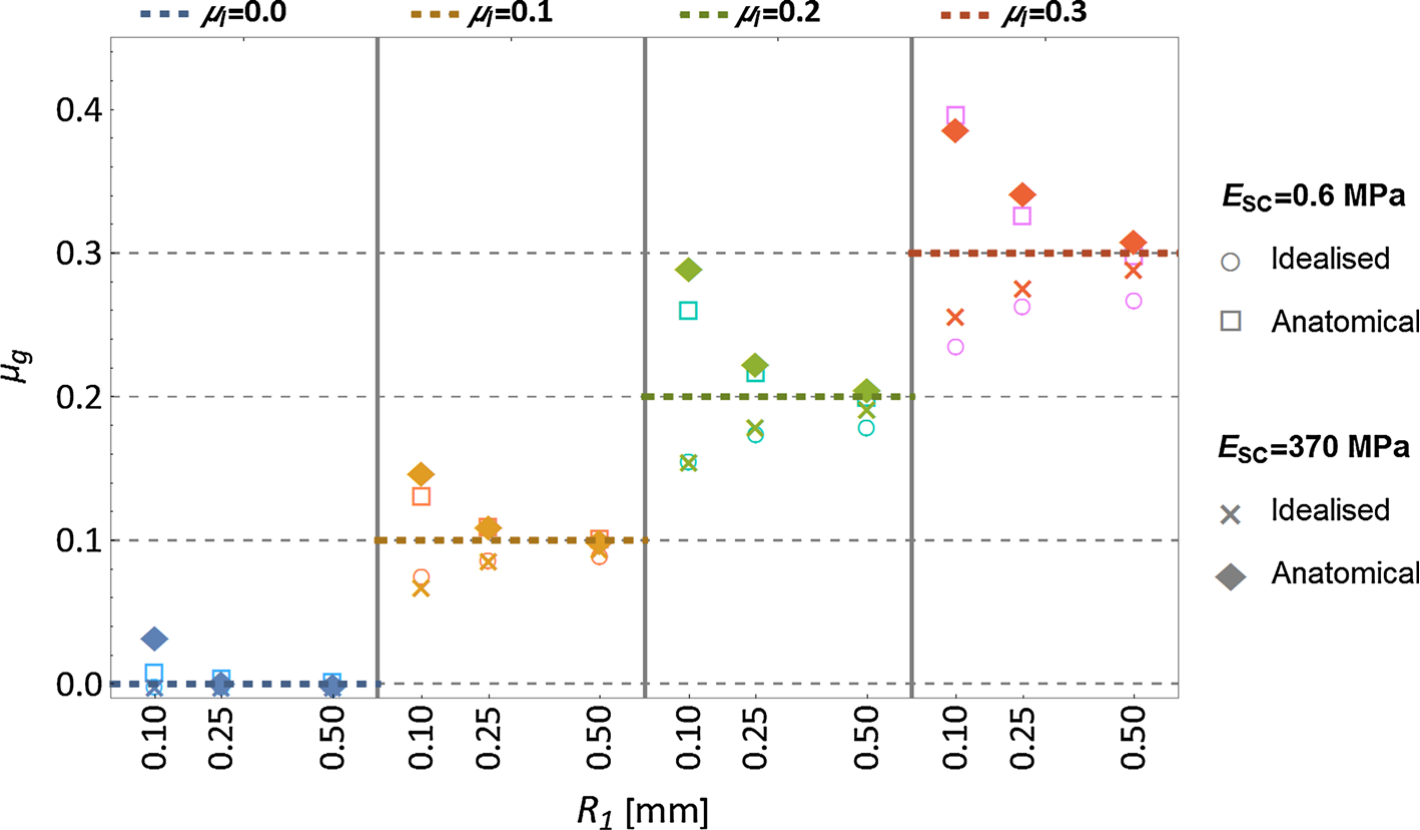
Global coefficient of friction *μ*_*g*_ determined from the sliding friction simulations as a function of indenter radius *R*_1_ and *stratum corneum* stiffness *E*_*SC*_, for the four contact interaction conditions specified with the local coefficient of friction *μ*_*l*_ (indicated by *coloured dashed lines*) (Color figure online)

**Fig. 5:**
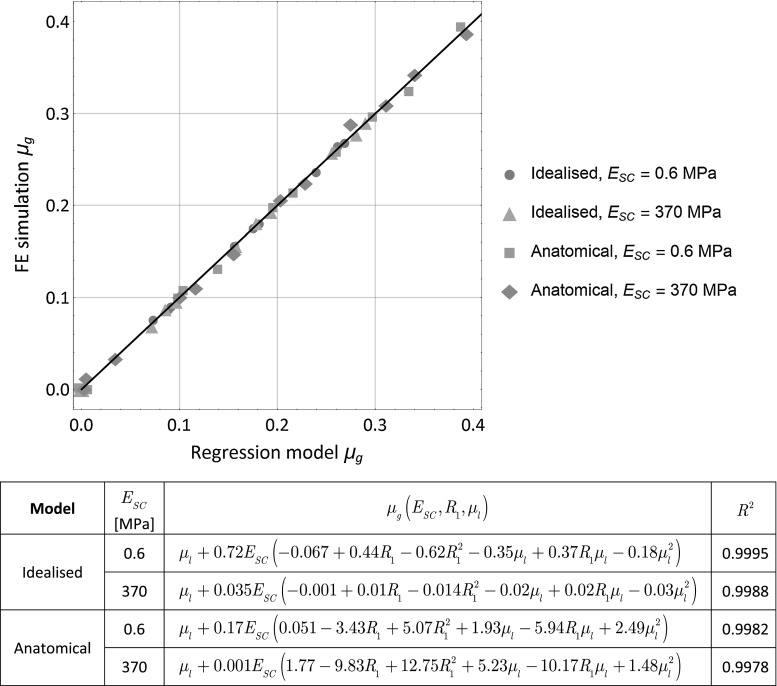
Correlation between the global coefficient of friction *μ*_*g*_ calculated by the regression model as a function of the *stratum corneum* stiffness *E*_*SC*_, indenter radius *R*_1_ and local coefficient of friction *μ*_*l*_, and the global coefficient of friction calculated from the finite element (FE) simulations

In Fig. 4, the global friction results are compared for both cases of *stratum corneum* stiffness (*E*_*SC*_ = 0.6 MPa and *E*_*SC*_ = 370 MPa), for each of the specified local friction conditions and for both idealised and anatomical models. In these results, the difference between the global and local coefficients of friction is clearly evidenced in most of the non-frictionless cases. For the idealised skin model, the global friction coefficient appears to be a fraction of the applied local friction coefficient, whereas this trend is reversed for the anatomical skin model. In that case, global friction is larger than local friction. There is also a clear correlation between indenter size and global friction coefficient. The analysis showed that the global coefficient of friction can be estimated with a regression model of the form:

7 $$\mu_{g} \left( {E_{SC} ,R_{1} ,\mu_{l} } \right) = \mu_{l} + c_{1} E_{SC} \left( {c_{2} + c_{3} R_{1} + c_{4} R_{1}^{2} + c_{5} \mu_{l} + c_{6} R_{1} \mu_{l} + c_{7} \mu_{l}^{2} } \right)$$

given that *δ* = *R*_1_/2, and where the constants {*c*_*i*_, *i* = 1.0.7} are dependent upon the type of model and the stiffness of the *stratum corneum.* This model provided a high correlation with the calculated global coefficients of friction, with a coefficient of determination *R* ^2^ > 0.997 for the different sets of results, for each type of model and *stratum corneum* stiffness (see Fig. 5). It is likely that the ratio of deflection with respect to the thickness of the *stratum corneum* as well as the geometrical characteristics of the skin topography play an important role on these parameters. So, this regression cannot be generalised to other conditions, mechanical and geometrical properties.

This nonlinear trend between the indenter size (i.e. indenting conditions) and the relative difference between the global and local friction coefficients is linked to the pressure distribution for each of the indenting conditions (i.e. *δ* = *R*_1_/2), in which a higher pressure was exerted by the largest indenter. In the idealised model simulations, the level of contact pressure was maintained constant during each sliding simulations. The indentation conditions of the anatomical model simulations were equivalent to those of the idealised model, under the assumption of a nominally flat surface. The trend was that with a smaller indenter radius, the global friction increased, and even though a larger pressure was applied to the skin surface by the *R*_1_ = 0.50 mm indenter, the simulations with the larger indenter led to a global coefficient of friction closer to the assigned local one.

In the frictionless cases, the idealised skin model, as expected, showed no resistance to motion with no amplification or reduction in the coefficient of friction from the microscopic to the macroscopic scale. In contrast, even for frictionless conditions, the anatomical model results indicated that the skin topography and its deformation were sufficient to induce macroscopic friction: *μ*_*g*_ = 0.004 and *μ*_*g*_ = 0.001 for, respectively, the soft (*E*_*SC*_ = 0.6 MPa) and hard (*E*_*SC*_ = 370 MPa) *stratum corneum*.

In the non-frictionless simulations, the anatomical and idealised skin models showed opposite response of global friction with respect to local friction. In the idealised model, the global friction coefficient exhibited an average reduction of 13.2% while an increase of 15.7% was observed for the anatomical model. For both cases, the stiffening of the *stratum corneum* (*E*_*SC*_ = 0.6 MPa to *E*_*SC*_ = 370 MPa) lead to an additional increase in the global coefficient of friction: 3.4% for the idealised model and 5.2% for the anatomical one. For both skin models and for the smallest indenter, a larger difference between the global and local coefficient of friction was found (Fig. 4).

In summary, the main findings highlighted in Fig. 4 are:

- *μ*_*g*_ ≤ *μ*_*l*_ for the idealised model and *μ*_*g*_ ≥ *μ*_*l*_ for the anatomical model.
- There is a correlation between the stiffness of the *stratum corneum* and the global coefficient of friction: they increase together.
- As the indenter size increases, the global coefficient of friction tends to the local one.

**Fig. 6:**
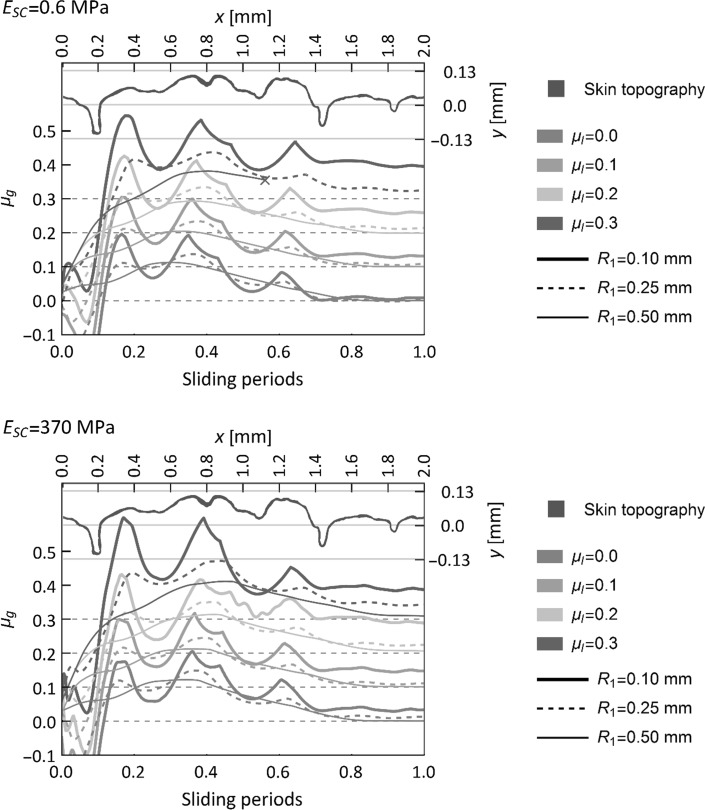
Evolution of the cumulative global coefficient of friction *μ*_*g*_ along the sliding path as the indenter slides over the skin surface (one sliding period). The geometry of the skin surface is layered over this plot to relate evolution of global friction and geometric features of skin microrelief (Color figure online)

The cumulative evolution of the local coefficient of friction along the sliding path using the integration procedure described in Sect. 2.3 is plotted in Fig. 6 for the anatomical models featuring a soft and harder *stratum corneum* and for each indenter size. The geometry of the skin was included in this plot, with respect to the models coordinate system (*x*, *y*), where *y* = 0 mm represents the mean height of the skin model, in order to identify the simultaneous effects of the skin topographic features and indenter radius on the global coefficient of friction.

It was observed that the cumulative (and not instantaneous) global coefficient of friction increased when in contact with the skin topographic protrusions. Such an increase was more significant for the simulations with the indenter of smallest radius (*R*_1_ = 0.1 mm), which despite being subjected to lower indentation depth, was more susceptible to interlocking with the skin microasperities. On the contrary, the global friction curve was smoother for the larger indenters as less interlocking took place. The relation between the skin topography and the global friction is evident in both models (*E*_*SC*_ = 0.6 MPa to *E*_*SC*_ = 370 MPa) cases, as the cumulative global coefficient of friction increases significantly when the indenter faces the highly protruding crests at sliding distance *x* = 0.1 mm, *x* = 0.6 mm and *x* = 1.1 mm.

## Discussion

Many physical experiments have proved the relevance of considering the surface topographic features of *solid materials* on the skin friction response [2], including textiles [7, 19, 63] and hard surfaces [17, 18]. Other studies have revealed that not only the surface roughness but also the asperity geometry is determining factors in the global friction response [4, 20, 23, 64]. The influence of the skin topography on its self-friction, however, has proved difficult to characterise. The effects of the skin surface topography on the friction response of skin have been called into question by Gerhardt et al. [19] in their study of skin–textile friction on young and aged people. Aged skin has rougher geometrical characteristics and stiffer *stratum corneum* than the younger one. These characteristics would suggest that the deformation component of friction is stronger than the adhesive one in aged skin, while the opposite response is expected in younger skin. Despite this, Gerhardt et al. [19] concluded that these two effects may balance themselves overall as they found no significant difference in the skin friction response between young and aged skin. Derler and Gerhardt [2] reviewed the literature of experimental work studying the link between the skin topography and its global friction, in which only two studies are referenced: contradicting results were provided by Egawa et al. [50], who showed that the skin surface roughness is a useful indicator for the prediction of the skin coefficient of friction when moisture was accounted for, but does not directly correlate with friction; Nakajima and Narasaka [24] showed that the density of the skin primary furrows, which is reduced with ageing, is correlated with skin friction. In ageing skin, parallel structural changes affect both its topography, internal structure and—if one focuses on linear elasticity—its Young’s modulus, raising questions about the nature and mechanisms of the interplay between furrow density and skin stiffness and their role on skin friction [2].

In our study, all of the anatomical simulations showed greater global friction than their idealised counterparts. This indicates that the global friction response is dominated by the resistance provided by the skin topographic features, which is one of the leading mechanisms of solid friction [45]. Naturally, it is important to keep in mind that these results are to be considered within the context of our modelling assumptions, namely that only mechanics is at play and that adhesive forces and humidity-induced volumetric changes in the *stratum corneum* are not *explicitly* accounted for.

As relative humidity increases, the stiffness of the *stratum corneum* can be reduced by several orders of magnitude [12, 39]. In a contact mechanics context, this phenomenon is potentially very significant as, under load, softening of the *stratum corneum* might increase contact area and, therefore, adhesive forces, increasing local and global frictional response. This response is also dependent on the surface energy of the contacting material. In our computational models, the different values assigned to the local (microscopic) coefficient of friction were set to represent different levels of local adhesion, as an interplay between humidity and surface energy of the contacting material [12], as well as the structural effect of deforming *nanoscopic* asperities averaged at the flat external surface of each finite elements forming the outer surface of the *stratum corneum* layer in our model.

Even though the *stratum corneum* layer was less than 10 µm thick, variation of its in stiffness had a visible effect on the skin global friction response (Fig. 4). As expected, in the case of a softer *stratum corneum*, the skin was subjected to higher deformation around the indenter, thus leading to larger real contact area. In our study, the effects of increase in adhesive response on contact pressure were not considered, and only geometrical effects were isolated and analysed. This explains how our simulations showed an increase in global friction with stiffer (i.e. dryer) *stratum corneum* while typically, skin friction tests report higher coefficients of friction with larger contact areas as higher adhesion resultant forces are produced [12].

Computational analyses showed that for the idealised skin model, the global coefficient of friction was smaller than the local one. This would suggest that the normal component of the reaction force at the indenter becomes dominant over the tangential component (*i.e.* decrease in the macroscopic coefficient of friction). It is believed that this effect is due to the way the idealised geometries of the skin layers deform in combination with the displacement-controlled sliding motion of the indenter. In frictionless conditions, this deformation is symmetrical so the forces experienced ahead and behind the indenter centre of mass balance each other, resulting in an effective frictionless global response. However, the local friction influences the drag and relative motion of the contacting surfaces: the surface of the skin tends to bend and “sinks in” vertically so to minimise the formation of a bow wave resisting lateral displacement. This effectively disrupts the balance of contact forces ahead and behind the indenter, resulting in a “push forward” effect that reduces the global coefficient of friction with respect to the local one.

In the anatomical skin model, the global friction measured resulted from the combined effects provided by the compliance of the contact (*i.e.* larger contact area), the resistance provided by the skin topographic and interfacial shear strength (provided by *μ*_*l*_ > 0.0). In frictionless contact, a global coefficient of friction of 0.004 and 0.001 (for soft and stiff *stratum corneum*, respectively) was obtained from the resistance provided by the skin topography only. These results provide a quantitative indication of the contribution of microasperity contact to the skin global friction and how the mechanical properties of the skin outer layer can influence these results. The disparity between the idealised and anatomical results provides insights into how topographic features of the skin can amplify the skin friction response, regardless of the local friction conditions.

The low stiffness of the *stratum corneum*, especially under high humidity conditions and plasticisation [23, 65] makes the skin response to deformation comparable to that of rubbers in the rubbery region [21, 27]. According to the theory of rubber friction developed by Persson [66], asperities of the soft rubbery material adapt to the contacting surface providing a smooth contact interface. Under this reasoning, many experimental tests assume the skin to be flat, considering that the real contact area is equal or close to the apparent one and overlooking any potential contribution of the skin topography to its global friction [12, 67]. In the present study, the characteristics of the contact interaction involved *localised* small deformation characteristic of micro- and nano-tribology studies [14, 28, 68], showing the relevance of the microasperity contact of skin in low-deformation contact (such as in contact interactions with clothing textiles). These effects have been implicitly captured in the experimental measurements of global coefficient of friction, but they had not been quantified until now. It is possible that at higher indentation loads, such as those observed during sitting or at foot soles or prosthetic interfaces, the external surface of the anatomical skin model would deform enough to provide a smooth contact interface. The effects of higher loads on the skin global friction response—where the role of the underneath skin layers and their inherent material inhomogeneities might also be more significant—remain to be explored in future investigations.

Apart from the Young’s modulus of the *stratum corneum*, no other humidity-dependent properties were considered in our simulations. Skin microclimate (*i.e.* relative humidity level and temperature) can modify the physico-chemical properties of the contact interface and lead to higher friction force via alteration of the interfacial shear strength [12] rather than solely via changes in contact area. Furthermore, water can be absorbed into the *stratum corneum* intercellular space, increasing the thickness of this outer layer up to three times for a 4-h water exposure [69]. Sopher and Gefen [11] implemented a finite element model of skin with a simplistic grooved topography. The model evaluated the combined effects of *stratum corneum* thickness, shear modulus and coefficient of friction at the skin–support interface on the shear stress distribution within the skin layers. In their study, they showed how these parameters can increase or reduce the risk of potential damage within the skin. Swelling of the *stratum corneum* would modify the skin microstructure, smoothing out skin crests and superficial furrows, and closing up the deeper furrows. Furthermore, the thickness of this outer layer would modify the overall skin elastic response as discussed earlier. So, it is likely that, if one takes into account the structural changes humidity causes in the *stratum corneum*, these changes could also significantly influence the global skin friction response. In Figs. 4 and 6, for the anatomical skin model, it is clear that the global friction can be significantly higher than the local one and this is based on the modelling assumption that only deformations modulate global friction. In the light of this observation, it can be conjectured that, compared to smooth skin, skin featuring a high degree of topographic roughness—as is the case in elderly subjects (e.g. deep wrinkles)—could be particularly prone to generate higher macroscopic friction if the *stratum corneum* stiffness is minimal.

In reality, the water-induced volume changes in the *stratum corneum* layer coupled to an increase in adhesive forces at the local level would likely lead to even larger increase in microscopic friction. The hypothesis that this could therefore be a plausible mechanism for the prevalence of skin tears in the elderly population [9] should be explored in future studies.

In the experimental analysis of skin and elastomers friction, interfacial adhesion is often considered to be the main contributor to global friction while deformation-induced friction (including viscoelastic effects) is thought to provide only a minor contribution [7, 12, 17, 22, 65]. Friction force is typically written as *f*_*μ*_:

8 $$f_{\mu } = f_{\mu }^{def} + f_{\mu }^{adh}$$

where *f*_*μ*_^*def*^ and *f*_*μ*_^*adh*^ are, respectively, the deformation-induced and adhesive component of friction force. Here, it is relevant to point out that Eq. (8) assumes that *f*_*μ*_^*def*^ and *f*_*μ*_^*adh*^ are uncoupled [70]. This assumption might be questionable depending on the nature and magnitude of several factors such as surface energies of the interacting surfaces, strain levels, microtopography and/or surface/bulk mechanical properties. Deriving an analytical model of friction valid for arbitrary geometry, constitutive models and strain/load levels would be a Titanesque—if not impossible—task because of our current lack of understanding of such a complex multiphysics phenomenon and the likely intractability of the resulting equations. In these circumstances, computational models can be very useful and complementary to physical experiments in enabling the study of complex coupled physics phenomena over complex geometric and material domains. In principle, a finite element model featuring the appropriate constitutive equations for surface physics and continuum mechanics would naturally account for the coupling of the physical processes responsible for friction, particularly with regard to surface deformations of complex microtopographic features.

In the analytical model developed by Wolfram [65], the adhesion-induced friction force is defined as:

9 $$f_{\mu }^{adh} = \tau A$$

where *A* is the contact area and *τ* the interfacial shear strength. In human skin, the interfacial shear strength is dependent on the applied contact pressure [12], meaning that for a given normal force any alteration of the contact area will modify the interfacial shear strength. In our model, the interfacial strength was specified with the definition of the local coefficient of friction *μ*_*l*_, but this parameter was set constant and independent of *A*. Consequently, the effect of reduction in global friction with larger contact area observed in our results is derived purely from deformation.

In the analytical model of Greenwood and Tabor [71], widely applied to the analysis of skin friction [12, 22, 51, 65, 72], the deformation component *f*_*μ*_^*def*^ was related to the level of deformation and viscoelastic dissipation effects (hysteresis) induced under the sliding indenter. In this model, hysteresis losses are derived from the elastic work required to move the indenter forward in a rolling motion. In the case of a sliding motion, additional work is provided by shearing losses. It must be emphasised that although Adams et al. [12] interpreted the deformation component of skin friction as a by-product of sub-surface viscous dissipation under the front of the slider, and purely elastic deformations also contribute to deformation-induced friction as demonstrated in the present computational study for the anatomical and idealised model. Elastic deformations of the skin layers and surface asperities effectively act as geometrical constraints that resist motions of the slider and therefore contribute to frictions through complex load redistribution mechanisms. These mechanisms are not exhibited for idealised geometries like those characteristic of the analytical model of Greenwood and Tabor [71] and of the idealised computational model presented in this study. For idealised rectilinear flat geometries featuring purely elastic materials interacting with an elastic slider trough frictionless contact, there is no dissipation within the bulk material; the contribution of the deformation component is null as the elastic work compressing the material ahead the indenter is counteracted by the elastic recovery behind the indenter [73]. This was also observed in the frictionless simulations shown in Fig. 4, where a negligible macroscopic friction was measured.

Although the constitutive model used for the skin layers was that of a conservative material (*i.e.* neo-Hookean elasticity), it is relevant to point out that the dissipative Newmark scheme was implemented to provide dynamic stabilisation to the highly nonlinear contact procedure. The effects of this procedure were sufficiently small to have a negligible effect on the solutions so that deformations of the skin could be considered fully recoverable.

The computational study presented in this paper demonstrated that local deformations of the skin microrelief and internal microstructure (i.e. complex geometry of the *stratum corneum* and underlying living epidermis and dermis as well as variations in mechanical properties of the *stratum corneum*) can provide a significant contribution to friction forces measured at a higher length scale. Moreover, it is clear that, for a given local friction value, the resulting global friction can be significantly different (e.g. in simulation with parameter combination [*E*_*SC*_ = 370 MPa, *R*_1_ = 0.10 mm, *μ*_*l*_ = 0.1], *μ*_*g*_ was 48% larger than *μ*_*l*_). This finding is of particular relevance to the modelling of contact interactions where global (*i.e.* macroscopic) coefficients of friction are often applied to systems featuring microscale interactions. For example, this was the case of a microneedle penetration model by Kong et al. [53] in which they applied the coefficient of friction obtained from the interaction of a 10-mm-diameter sphere on volar skin to the contact interactions of microneedles with skin. In these circumstances, the differences between global and local coefficient of friction could have significant effects on the output results, as shown in computational friction sensitivity analyses conducted by Sopher and Gefen [11] and Oomens et al. [74].

To the best of our knowledge, the bridging mechanisms between the local and global friction response *of skin* have yet to be experimentally quantified. Broitman [75] compared the tribological properties of fullerene-like carbon nitrite films in macro-, micro- and nano-scales friction tests, providing an example of *solid coatings* materials in which the coefficient of friction can vary from 0.05 to 0.3 depending on the spatial scale considered. Such differences are the result of scale-dependent physical interaction mechanisms, such as multiasperity interlocking, localised deformation and/or small-range molecular forces. Macroscopic friction tests are unlikely to discriminate between nano- to microscopic friction effects.

In their computational homogenisation study, Stupkiewicz et al. [49] considered first a simple plane strain configuration consisting of a smooth compliant hyperelastic half-space sliding over an idealised sinusoidal rigid surface. The effects of height asperity and local friction on the resulting computed homogenised global friction were assessed. It was shown that the resistance provided by surface topographic features significantly influences the global friction response of the contacting materials, not only in the magnitude of the global frictional response but also by causing anisotropic friction. A similar approach was taken by Temizer [76], who recently developed a robust 3D computational contact mechanics framework based on isogeometric finite element techniques to study soft matter friction. The influence of microscopic roughness on the macroscopic coefficient of friction for boundary layers was investigated using this numerical toolbox.

Like in the studies of Stupkiewicz et al. [49] and Temizer [76], the computational homogenisation procedure proposed in the present study is not restricted to linear material and kinematics. It can cope with nonlinear materials and finite deformations and, because it is based on finite element techniques, can accommodate geometrical domains of nearly arbitrary complexity. This offers a significant advantage over analytical models which quickly break down or become intractable for nonlinear behaviour or non-idealised geometries. It is the authors’ opinion that the research methodology presented in this paper has proved very efficient in gaining a mechanistic insight into skin friction while also paving the way for more advanced physics-based studies which will take us even further in our understanding of soft matter friction in general and skin friction in particular.

The load dependency of the skin coefficient of friction has been widely documented. In their review of tribology of skin, Derler and Gerhardt [2] related this phenomenon to the area of contact and adhesive friction. The relation between contact area and interfacial shear strength has been demonstrated by Kwiatkowska et al. [22] as a larger coefficient of friction was experimentally measured for a glass indenter with a larger diameter and identical loading conditions. A systematic and thorough investigation of this effect is left for future studies.

The increase in friction as a result of the resistance provided by the skin topographic features is illustrated in Fig. 6. In the *anatomical* model, higher values of global friction were induced when the indenter encountered a skin crest. These high values are compensated by drastic reduction in friction when the indenter passes beyond the crests.

The anatomical model used in the present study is a first step towards a more advanced model in which the 3D geometry of the skin microstructure will be taken into account. At the moment, the model captures the 2D detailed geometry of the skin layers as well as that of the microscopic asperities of the skin surface. The model was simplified with a number of assumptions that offer the benefit of faster runtimes for simulations: plane strain analysis, no time-dependent effects as well as isotropy and homogeneity of the materials in each layer. The most obvious limitation of the model stems from the restriction to two spatial dimensions. In a 3D setting, the reaction forces at microasperity contact are expected to span multiple directions (not only orientated along the sliding direction, as in the 2D case) which might alter the skin global friction response so that anisotropic friction is produced. Within a 3D modelling environment, more realistic aspects of skin micromechanics can be taken into account such as skin collagen fibre architecture and anisotropic elasticity which are likely to produce more complex strain and stress distributions.

In the present model, the skin layers were modelled as isotropic neo-Hookean hyperelastic materials. One of the principal mechanical characteristics of skin is its anisotropy and stiffening behaviour in tension. Lanir and Fung [77] documented these characteristics on rabbit skin; their results have been widely used for the validation of entropic-based constitutive models developed to represent the anisotropy of skin in general and that of dermal tissue in particular [78–80]. In the current 2D plane strain modelling approach and for the loading and boundary conditions enforced, the stretch-stiffening anisotropic characteristics provided by the dermis are not relevant, but they should be considered for future simulations involving larger deformations and the analysis of contact interactions in 3D.

Furthermore, incorporation of skin time-dependent behaviour (*i.e.* viscoelasticity) would allow to extend computational analyses to account for the effects that sliding velocity and material dissipative processes have on the global friction response of skin. Persson [81] demonstrated the relevance of these effects in the context of rubber friction while Tang and Bhushan [28], Tang et al. [82] and Adams et al. [12] made similar observations for skin friction.

In their experimental study, Johnson et al. [83] related the coefficient of friction of skin to sliding velocity using a power-law expression, but the effects of viscoelastic skin relaxation on the skin asperities, likely to be affected by the sliding velocity, were not investigated [2]. Further effects on the skin mechanical response (deformation and global friction) can be expected by accounting for in vivo pre-strains in the dermal tissue, which provide a stiffer substrate to the *stratum corneum* [40]. In future studies, we aim to refine and implement these microstructural and material characteristics in order to further and deepen our understanding of their interplay and individual role in relation to skin tribology.

## Conclusion

In this study, the role of the skin microscopic surface topography and internal microstructure in conditioning the deformation component of macroscopic friction was investigated using a finite element homogenisation procedure. The modelling methodology and associated simulation tools go beyond what current analytical models of skin friction can offer: the ability to accommodate nonlinear kinematics (i.e. finite deformations), nonlinear constitutive properties and the complex geometry of the skin layers. It was demonstrated that this approach offered a new level of mechanistic understanding into how local skin deformations in combination with alterations in the mechanical properties of the *stratum corneum* alone can modulate global friction and lead to a global friction significantly different than the local one.

From this study, it is concluded that in the deformation component of skin friction (i.e. ignoring the relation between real contact area and adhesive friction):

- The global coefficient of friction can be significantly different to the local coefficient of friction. The difference between the micro- and macroscopic friction is dependent on the microscopic geometry of both contacting surfaces, i.e. skin and rigid counterpart, as well as the mechanical properties and geometry of the skin layers.
- The global coefficient of friction is lower than the local one when the skin is assumed to be flat. Although this effect seems to be counter-intuitive, it was already observed in previous works, e.g. [50].
- The global coefficient of (deformation-induced) friction is magnified by the structural effects engendered by the geometry of skin microasperities. The presence and deformations of these asperities lead to interlocking with the rough contacting surface.
- The mechanical properties of the *stratum corneum* can considerably modify the global friction response by influencing the local skin deformation at the contact area and providing stiffer asperities that increase resistance to motion, and therefore, the global friction response.

The modelling approach proposed here should be viewed as complementary to physical experimental protocols characterising skin friction as it may facilitate the interpretation of observations and measurements and/or could also assist in the design of new experimental quantitative assays. These results also suggest that care should be taken when assigning a coefficient of friction in computer simulations, as it might not reflect the conditions of macroscopic friction one intend to represent. The findings of this computational study could be significant for a wide range of applications where skin friction is relevant, from superficial pressure ulcers, through male/female shaving and cosmetics to automotive safety.

## Electronic supplementary material

Below is the link to the electronic supplementary material.

Supplementary material 1 (DOCX 650 kb)
