# Supplementary material for: Skin Microstructure is a Key Contributor to Its Friction Behaviour
Source: Tribol Lett. 2016 Nov 30;65(1):12. doi: 10.1007/s11249-016-0794-4 (PMC6961497; doi:10.1007/s11249-016-0794-4)
Supplement: Supplementary file 1 — Supplementary material 1 (DOCX 650 kb) [file 11249_2016_794_MOESM1_ESM.docx]

**Skin microstructure is a key contributor to its friction behaviour.**

Maria F. Leyva-Mendivil^1,2^, Jakub Lengiewicz^3^, Anton Page^4^, Neil W. Bressloﬀ^5^ and Georges Limbert^1,2,6 *^

^1^national Centre for Advanced Tribology at Southampton (nCATS), Faculty of Engineering and the Environment, University of Southampton. Southampton, SO17 1BJ, UK.

^2^Bioengineering Research Group, Faculty of Engineering and the Environment, University of Southampton. Southampton, SO17 1BJ, UK.

^3^Institute of Fundamental Technology Research, Polish Academy of Sciences (IPPT PAN), ul. Pawinskiego 5B; 02-106 Warszawa, Poland

^4^Biomedical Image Unit, Faculty of Medicine, University of Southampton, Southampton General Hospital, Southampton, SO16 6YDJ, UK.

^5^Computational Engineering and Design Group, Faculty of Engineering and the Environment, University of Southampton. Southampton, SO17 1BJ, UK.

^6^Laboratory of Biomechanics and Mechanobiology, Division of Biomedical Engineering, Department of Human Biology, Faculty of Health Sciences, University of Cape Town, Observatory 7935, South Africa.

**Journal:** Tribology Letters

**Article type:** Research Article

**Date:** 9 September 2016

***Corresponding author:**  Tel: +44 (0)2380 592381; fax: +44 (0)2380 593016.

E-mail address: [g.limbert@soton.ac.uk](mailto:g.limbert@soton.ac.uk)

# Supplementary material: validation of the indentation simulations against line contact theories

This supplementary material describes the analytical verification of the finite element simulations of contact interactions of a cylindrical or discoidal body with a half-space. The validation was performed by comparing analytical solutions of contact area and total deflection against the results obtained in the finite element simulations for various contact theories found in the literature. This validation was required for the progression of an idealised model of skin as a flat homogeneous material in contact with a rigid indenter, into an anatomical multi-layer model of human skin subjected to sliding contact interactions.

The analytical model to obtain the contact area was derived from the Hertzian theory of contact between two cylinders with parallel axes with equal length $L$. The first cylinder of radius $R_{1}$ represented the discoidal indenter. The second cylinder of radius $R_{2}=\infty$ mm represented the skin material as a half space. The indenting force of magnitude$F_{N}$, normal to the skin surface, is uniformly distributed along the length of the indenter cylinder. This can be represented in 2D as a punctual load of magnitude$P=F_{N}/L$. Under these conditions, the contact area is described by a $2b\times L$ rectangle, where the half width $b$ is the distance between the indenter axis and the further region in contact, as projected over the skin surface (see **Fig. 1**). As $L>>2b$, these contact conditions are known as ‘line contact’, for which the half width $b$ is the most representative parameter of the contact area.

The half width $b$ for line contact is defined by the **Hertz theory of contact** as:

$$b=\sqrt{\frac{4R^{*}F_{N}}{\pi LE^{*}}}$$

where $R^{*}$ and $E^{*}$ are the reduced radius and reduced Young's modulus respectively. The reduced radius is defined in terms of the radii of the contact parts:

$$\frac{1}{R^{*}}=\frac{1}{R_{1}}+\frac{1}{R_{2}}$$

that, by considering$R_{2}\to\infty$, results in$R^{*}=R_{1}$. The reduced Young's modulus is defined in terms of the elastic properties of the materials in contact as:

$$\frac{1}{E^{*}}=\frac{1-\nu_{1}^{2}}{E_{1}}+\frac{1-\nu_{2}^{2}}{E_{2}}$$

where sub-indexes 1 and 2 refer to the indenter and flat materials, respectively. Assuming that $E_{1}\to\infty$ for a rigid indenter, $E^{*}=E_{2}/1-\nu_{2}^{2}$.

In response to the indenting force applied, the surface of the flat material is deflected a distance $\delta$from its original surface. Hertzian theory can be used for the estimation of maximum deflection in line contact between two cylinders with parallel axes as [1]:

$$\delta_{H}=0.319\left(\frac{F_{N}}{LE^{*}}\right)\times\left[\frac{2}{3}+\text{Ln}\left(\frac{4R_{1}R_{2}}{b^{2}}\right)\right]$$

where the subscript index *H* stands for Hertzian theory of contact. However, this equation cannot capture $\delta$ when one of the cylinders is represented with an infinite radius (*i.e.* a half-space) [2], as $\lim\delta_{R_{2}\to\infty}=\infty$. A great variety of models have been proposed in the literature for the estimation of $\delta$, for which a review is presented next, identifying each model with a Roman number subscript index.

Norden [3] provided an in-depth review of various models for contact of cylinders with a half-space. One of these models corresponds to the expression obtained from the work of Thomas, Hoersch [4] and Love [5]:

$$\delta_{I}=\frac{F_{N}}{L\pi E^{*}}\left[1+\text{Ln}\left(\frac{L^{3}\pi E^{*}}{F_{N}R^{*}}\right)\right]$$

which, although expressed in a different arrangement of parameters, is coincident with the work of Lundberg [6] and Puttock, Thwaite [2]. Another model corresponds to the work of Ferguson [7], considering an elliptical distribution of pressure on the plane surface:

$$\delta_{II}=\frac{F_{N}}{L\pi E^{*}}\left[-0.608205+1.5\text{ Ln}\left(\frac{L^{3}\pi E^{*}}{F_{N}R^{*}}\right)\right]$$

From the formulation for deflection of one half of a cylinder compressed by two diametrical forces [8], the deflection between two parallel cylinders can be derived as:

$$\delta_{III}=\frac{F_{N}}{L\pi E^{*}}\left[\text{Ln}\left(\frac{4R^{*}L\pi E^{*}}{F_{N}}\right)-1\right]$$

which is equivalent to the solution presented by Nakhatakyan [9]. Johnson [8] also presented a model for 2D contact between a cylinder and a half-space, estimating the deflection of a plane surface with respect of a point located directly underneath the indenter contact point at a distance $\zeta$ as:

$$\delta_{IV}=\frac{F_{N}\left(1-\upsilon_{2}^{2}\right)}{L\pi E_{2}}\left[2\text{ Ln}\left(\frac{2\zeta}{b}\right)-\frac{\upsilon_{2}}{1-\upsilon_{2}}\right]$$

which, assuming the indenter is much stiffer than the half-space material (*i.e.* $E_{1}>>E_{2}$), it is equivalent to the total deflection. It was indicated that $\zeta$ can be used to indicate the depth $h_{s}$ of the half-space in the model, as it does affect the contact interaction. Considering the thickness of the flat material, Johnson [8] reviewed the Wrinkler *elastic foundation* model. This model represents the flat material as an elastic foundation resting on a rigid surface instead of a half-space. The elastic response of this material is dependent on the displacement normal to the surface and excludes any shear response. Such behaviour is analogous to a mattress whose deformation is given by the resistance of its springs. With the Wrinkler model, the 2D contact between a cylinder and the `mattress' material can be approximated as:

$$\delta_{V}=\frac{3F_{N}}{4L\left(1.18E^{*}\right)}$$

The last model presented in this section, applied in the analysis of contact in roller bearings, reviewed by Harris, Kotzalas [10], accounts for the models from Lundberg [6] for line contact with semi-cylindrical stress distribution at the contact area:

$$\delta_{VI}=\frac{2F_{N}\left(1-\upsilon_{2}^{2}\right)}{L\pi E_{2}}\text{Ln}\left[\frac{L^{2}\pi E_{2}}{F_{N}\left(1-\upsilon_{2}^{2}\right)}\right]$$

All these models (Equations to ), were developed under the assumptions of Hertzian theory [2,3,8,10]:

1. the materials are linear elastic
2. the contacting surfaces are perfectly smooth and frictionless
3. loading is applied normal to the contact surface, neglecting shear effects
4. the area of contact is much smaller than the surface dimensions of both contacting bodies
5. strain levels are small and within the elastic regime

The deflection models (Equations to ) were evaluated by means of a sensitivity analysis considering variation of the stiffness of the flat material (*i.e.* skin) $E_{2}$, the indenter radius $R_{1}$ and displacement of the indenter$D_{y}$(inducing the skin deflection). The design of computational experiment was built using a normalised pseudo-random space filling sampling plan generated using a Sobol sequence from the Intel® Math Kernel Library (MKL) generator through Mathematica® (Wolfram Research, Inc., Champaign, IL, USA). The Sobol sequence is a low-discrepancy sequence that provides a uniform distribution of values for random sampling generation [11]. A fixed seed value was specified in order to maintain the same sequence of pseudo-random values in subsequent analyses. Three sets of 25 experiments, identified as a *sensitivity analysis* (SA) were generated to assess the effects of different levels of indentation and stiffness of the flat material. Each experiment was obtained by variation of the parameters within the ranges specified in **Table 1**.

Given the number of analytical deflection models, it was required to determine which of these models could appropriately capture the skin deflection when the skin is represented as flat homogeneous material. It should be noted that, despite the maximum imposed indenter displacement being $D_{y}=0.5$ mm, the maximum deflection captured in the sensitivity analysis simulations was $\delta=0.42$ mm. Models $\delta_{III}$ and $\delta_{V}$ showed a large difference from the simulations results, while higher agreement was shown for models $\delta_{I}$, $\delta_{II}$, $\delta_{IV}$ and $\delta_{VI}$, especially for $\delta<0.1$ mm (**Fig. 2**). At such a small deflection ($\delta<0.1$ mm), model $\delta_{IV}$ showed a mean and median error of 5.43% and 5.48%, respectively, and an interquartile range of 0.007. This model was of higher accuracy (lower error and lower dispersion) for the conditions applied in these simulations.

**Table 2** shows the relative error comparison between the six models evaluated and the finite element results. The variation observed among the analytical deflection models results reflect the different conditions considered in their development. In these simulations, where the height of the skin model was considered to be $h_{s}=5$ mm, the analytical model $\delta_{IV}$ showed a better correspondence to the finite element model results.

For the validation of the model, the half width $b$ and deflection $\delta$ measurements in the finite element simulations (AceGen/AceFEM) were compared to those predicted by the analytical models (Equations for half width, and for deflection). Results of the sensitivity analyses are depicted in **Fig. 3**. There was a good agreement between the analytical and numerical solutions, with a mean relative error of 4.3% for the half width, and 5.43% for the deflection which were considered sufficient to consider the idealised finite element model validated. These preliminary validation steps of the computational contact framework were essential to ensure a sound basis for further advanced contact analyses of the anatomically-based multi-layer skin models. These models featured a very complex geometry which prevented the derivation of any tractable analytical solution.

**References**

1. Stachowiak, G., Batchelor, A.W.: Engineering Tribology. In. Butterworth-Heinemann, (2013)

2. Puttock, M.J., Thwaite, E.G.: Elastic Compression of Spheres and Cylinders at Point and Line Contact. National Standards Laboratory Technical Paper **25**(25) (1969).

3. Norden, B.N.: On the compression of a cylinder in contact with a plane surface. In., pp. 73--243. Washington, D. C., (1973)

4. Thomas, H.R., Hoersch, V.A.: Stresses due to the pressure of one elastic solid upon another with special reference to railroad rails : a report. In. University of Illinois. Engineering Experiment Station. Bulletin; no. 212, (1930)

5. Love, E.R.: Compression of elastic bodies in contact. In. Defense Standards Laboratory, Australia., Australia, (1942)

6. Lundberg, G.: Elastische Berührung zweier Halbräume. Forschung auf dem Gebiete des Ingenieurwesens **10**(5), 201-211 (1939). doi:10.1007/BF02584950

7. Ferguson, B.: Elastic Deformation Effects in Precision Measurement. Microtecnic **11**, 256-258 (1957).

8. Johnson, K.L.: Contact Mechanics. Cambridge University Press, Cambridge, UK. (1985)

9. Nakhatakyan, F.G.: Precise solution of Hertz contact problem for circular cylinders with parallel axes. Russian Engineering Research **31**(3), 193-196 (2011). doi:10.3103/S1068798X11030208

10. Harris, T.A., Kotzalas, M.N.: Rolling bearing analysis. Essential concepts of bearing technology, 5th ed. CRC/Taylor & Francis, Boca Raton; London (2007)

11. Sobol, I.M.: On the distribution of points in a cube and the approximate evaluation of integrals. USSR Computational Mathematics and Mathematical Physics **7**(4), 86-112 (1967). doi:10.1016/0041-5553(67)90144-9

**Table 1.** Parameter limits for the analyses of sensitivity of half width and deflection sensitivity of the analytical models, with the use of the idealised skin contact model. $E_{2}$ - Young’s modulus of the flat material

| Analysis | Limits | $E_{2}$ | $R_{1}$ | $D_{y}$ |
| --- | --- | --- | --- | --- |
|  |  | [MPa] | [mm] | [mm] |
| SA 1: Large indentation | Min | 0.01 | 0.05 | 0.01 |
|  | Max | 400.0 | 0.5 | 1.0 |
| SA 2: Small indentation | Min | 0.01 | 0.05 | 0.01 |
|  | Max | 400.0 | 0.5 | 0.05 |
| SA 3: Low stiffness of flat material | Min | 0.01 | 0.05 | 0.01 |
|  | Max | 1.0 | 0.5 | 0.5 |

**Table 2** Error analysis between the analytical deflection models and the finite element (FE) results. Mean and median values of the relative error distribution for deflection $\delta<0.1$ mm are compared, showing the interquartile range (IQR) as a measure of error dispersion. Lower error and dispersion was found in model $\delta_{IV}$, as marked in bold font

| **Model** | **Mean error [%]** | **Median error [%]** | **IQR** |
| --- | --- | --- | --- |
| FE | 0.0 | 0.0 | 0.0 |
| $\delta_{I}$ | 23.05 | 23.17 | 0.0315 |
| $\delta_{II}$ | 6.30 | 5.92 | 0.0672 |
| $\delta_{III}$ | 53.05 | 52.88 | 0.1056 |
| $\delta_{IV}$ | **5.43** | **5.48** | **0.0070** |
| $\delta_{V}$ | 80.35 | 80.25 | 0.0273 |
| $\delta_{VI}$ | 10.78 | 9.26 | 0.1032 |


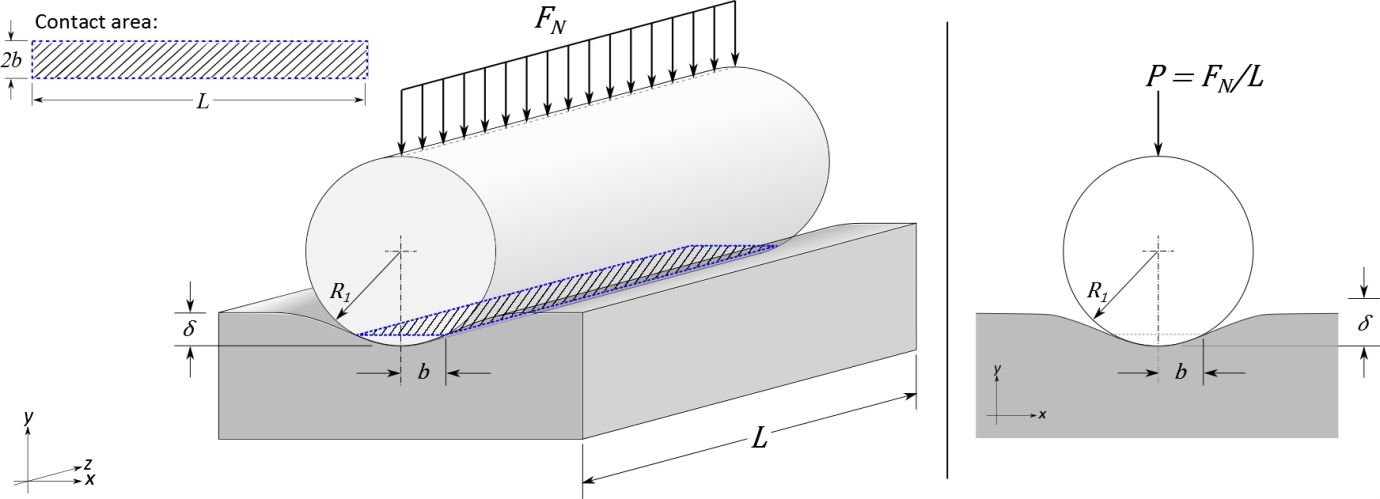


**Fig. 1** Line contact between a cylinder and a flat surface. The contact between a cylinder of radius $R_{1}$ and a half-space providing a flat surface, both of length $L$ in the $x$ direction, under indentation force of magnitude $F_{N}$, equally distributed along the cylinder length, causes the flat material to deflect a distance $\delta$, resulting in a contact area of $2b\times L$, where $b$ is the half width of the line contact area. This system can be simplified in 2D under the assumption of plane strain (infinitely long cylinders), where indentation is caused by a punctual load of magnitude $P$ (right)

| 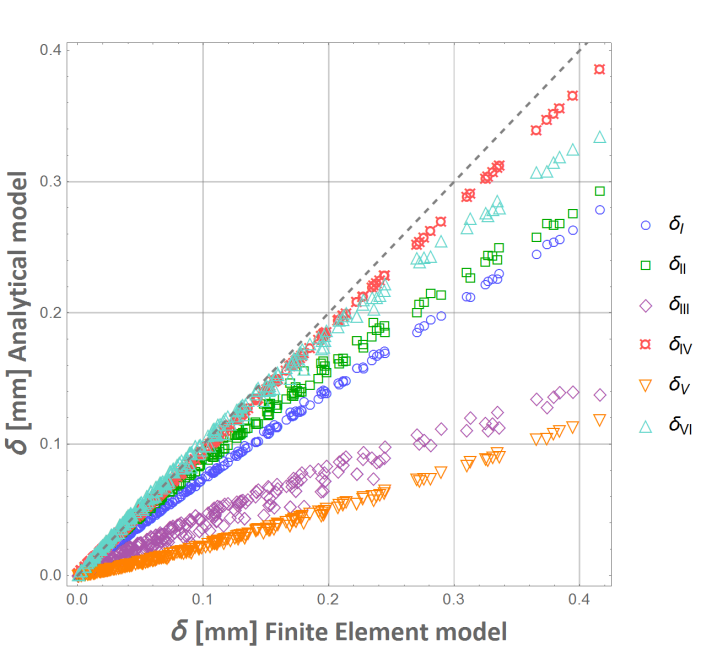 | 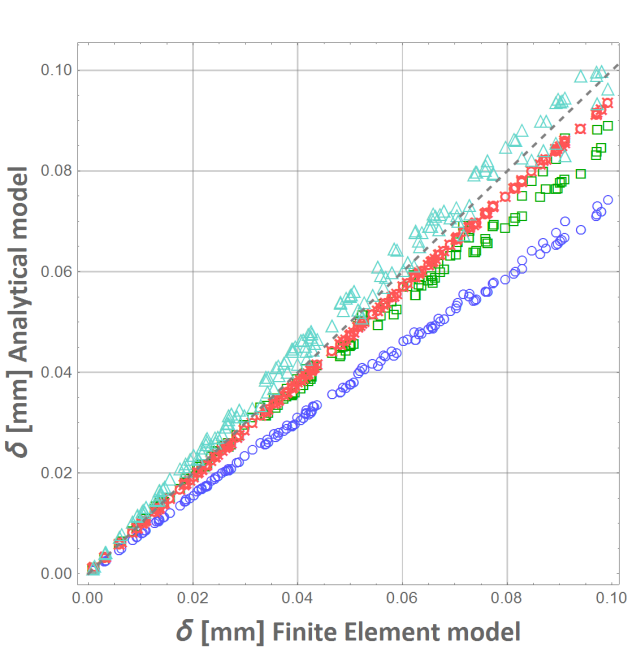 |
| --- | --- |

**Fig. 2** Analysis of the analytical deflection models. The deflection results of the finite element (FE) model simulations were compared to the results of the analytical models $\delta_{I}$ to $\delta_{VI}$ a) the full range of deflection observed in the simulations, and b) for $\delta<0.1$ mm, focusing on the models with closer match to the ideal fit (dotted line) which indicates a perfect fit between theory and computations

| 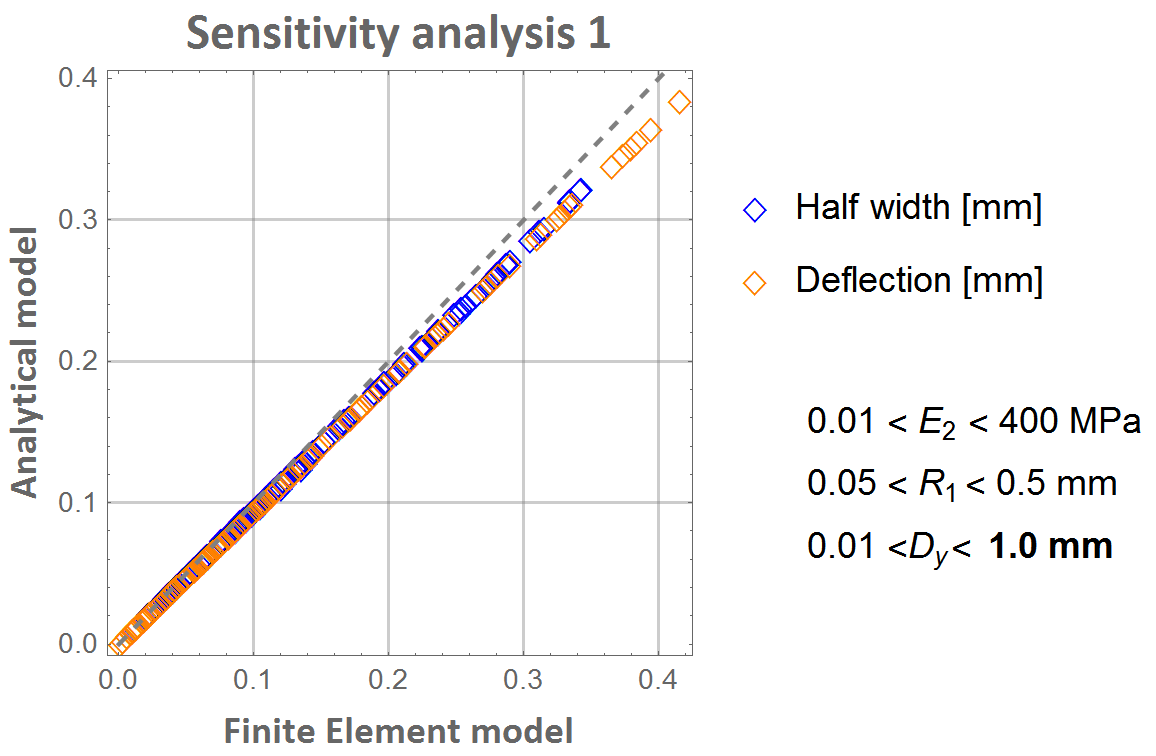 |
| --- |
| 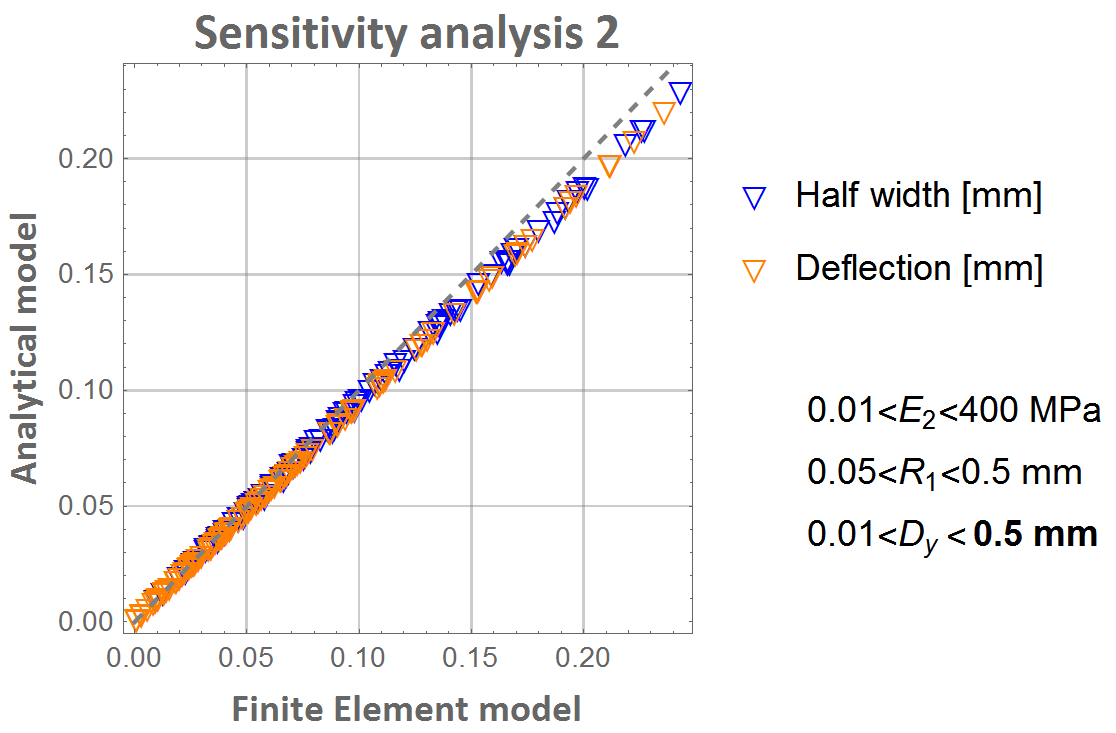 |
| 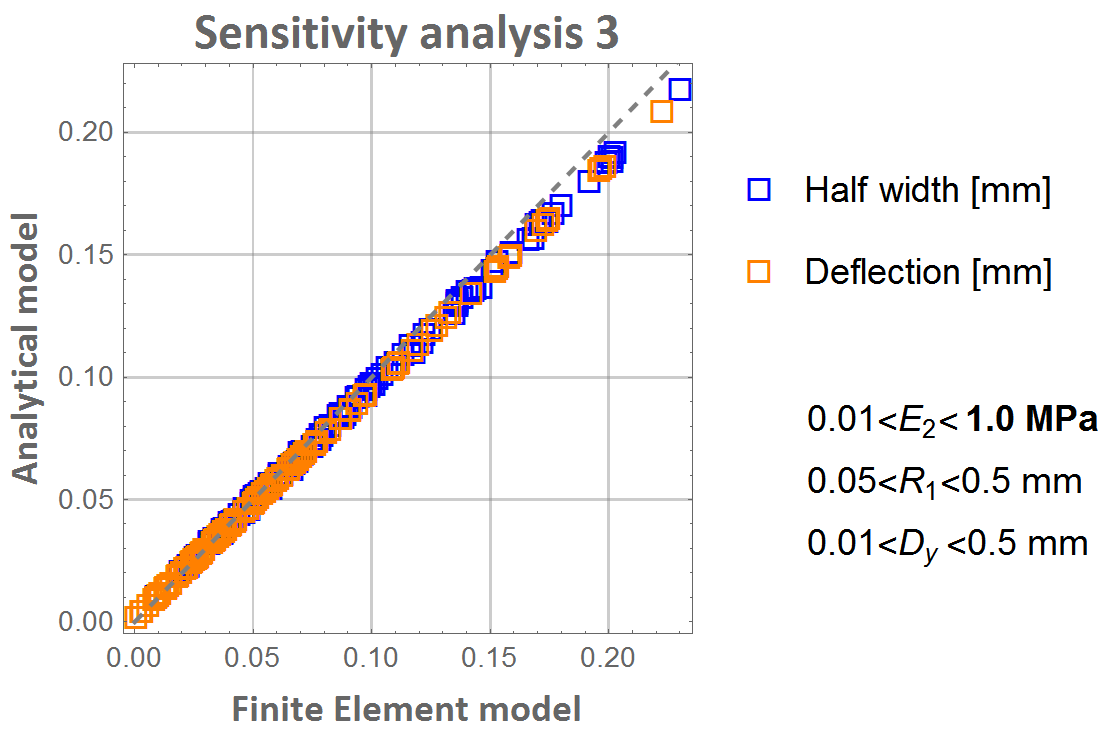 |

**Fig. 3** Fitting of the sensitivity analysis results to those of the analytical models for the calculation of half width, and deflection, with variation of the flat material stiffness$E_{2}$, indenter radius $R_{1}$ and imposed displacement$D_{y}$
